# A predictive model for catheter-related bloodstream infection in neonates with peripherally inserted central catheter

**DOI:** 10.3389/fmed.2025.1665068

**Published:** 2025-10-21

**Authors:** Fan-ying Zeng, Wen-yan Li, Hong-yun Ye, Chun-lian Xie, Hai-li Zhong

**Affiliations:** ^1^Department of Intravenous Catheter Care Outpatient Clinic, Ganzhou Women and Children's Health Care Hospital, Ganzhou, Jiangxi, China; ^2^Department of Nursing, Ganzhou Women and Children's Health Care Hospital, Ganzhou, Jiangxi, China; ^3^Department of Neonatal Intensive Care Unit (NICU), Ganzhou Women and Children's Health Care Hospital, Ganzhou, Jiangxi, China

**Keywords:** neonatal intensive care unit, peripherally inserted central catheter, catheter–related bloodstream infection, influencing factors, prediction model

## Abstract

**Objective:**

To explore the influencing factors of catheter-related bloodstream infection (CRBSI) in neonates with peripherally inserted central catheter (PICC) in the neonatal intensive care unit (NICU).

**Methods:**

A total of 200 neonates who underwent PICC placement were selected. They were randomly divided into a training set (*n* = 140) and a validation set (*n* = 60) at a ratio of 7:3. Clinical data of the neonates were collected, including general information, catheterization-related indicators, laboratory indicators, and other relevant indicators. Univariate analysis and multivariate Logistic regression analysis were used to screen the independent risk factors for CRBSI. The random forest algorithm was used to rank the importance of the risk factors, and the variance inflation factor (VIF) was used for multicollinearity diagnosis. A nomogram prediction model was constructed based on the independent risk factors. The predictive efficacy of the model was evaluated by receiver operating characteristic (ROC) curve, calibration curve, and decision curve analysis (DCA).

**Results:**

In the training set, 32 cases (22.86%) developed CRBSI, and in the validation set, 14 cases (23.33%) developed CRBSI, with no statistically significant difference (*P* > 0.05). Multivariate analysis showed that the age at catheterization, number of punctures, white blood cell count, number of days of antimicrobial use, and number of days of parenteral nutrition were independent risk factors for CRBSI (all *P* < 0.05), and the 5-min Apgar score was an independent protective factor for CRBSI (*P* < 0.05). The C-indexes of the nomogram model in the training set and the validation set were 0.923 and 0.881, respectively. The ROC curve showed that the area under the curve (AUC) in the training set was 0.921 (95% *CI*: 0.819–1.000) and in the validation set was 0.880 (95% *CI*: 0.768–0.992). The sensitivity and specificity in the training set were 0.909 and 0.844, respectively, and in validation set were 0.857 and 0.857, respectively.

**Conclusion:**

The nomogram prediction model constructed based on the screened independent risk factors can effectively predict the risk of CRBSI in neonates with PICC in the NICU, providing a basis for the clinical early identification of high-risk neonates and the formulation of preventive measures.

## Introduction

In the Neonatal Intensive Care Unit (NICU), the Peripherally Inserted Central Catheter (PICC) has become an important means of clinical treatment as it can provide long-term and safe venous access for critically ill newborns (1). However, Catheter-Related Bloodstream Infection (CRBSI), as one of the most serious complications of PICC, not only prolongs the hospitalization time of children and increases medical costs but may also lead to severe consequences such as sepsis and septic shock, and even threaten the lives of newborns (2). Data shows that the incidence of PICC-related CRBSI in the NICU is ~5%−20%. Due to the imperfect development of the immune system and the complex and changeable conditions of newborns, especially premature infants, their infection risk is significantly higher than that of other populations (3). Currently, the clinical prevention and control of CRBSI mainly rely on standardized nursing measures, such as aseptic catheterization and regular catheter maintenance. However, such measures often fail to cover all risk links (4, 5). In addition, the clinical manifestations of CRBSI in newborns lack specificity and are difficult to identify in the early stage, resulting in some children not receiving timely intervention and affecting the prognosis (6). Therefore, how to accurately identify high-risk children with CRBSI and construct a scientific prediction model to guide early clinical intervention has become a key issue in infection prevention and control in the NICU (7). In recent years, with the development of evidence-based medicine, the application of multi-factor prediction models in infection risk assessment have become increasingly widespread (8). By integrating multi-dimensional data such as the clinical characteristics of newborns, catheterization operation factors, and laboratory indicators, constructing a prediction model is expected to achieve a quantitative assessment of CRBSI risk. This study aims to explore the influencing factors of CRBSI in children with PICC in the NICU, construct a prediction model based on multi-dimensional indicators and verify its efficacy, providing a basis for early clinical warning and personalized prevention, thereby reducing the incidence of CRBSI and improving the prognosis of newborns.

## Materials and methods

### Study population

A total of 200 children who underwent PICC catheterization in the NICU of our hospital from January 2022 to December 2024 were selected. Inclusion criteria: (1) Gestational age at birth ≤ 42 weeks; (2) First PICC catheterization; (3) Informed consent was obtained from the parents of the children and they signed the consent form. Exclusion criteria: (1) Congenital immunodeficiency diseases; (2) Bloodstream infection was clearly present before catheterization; (3) Those who transferred to other hospitals or gave up treatment midway. The patients were randomly divided into a training set (*n* = 140) and a validation set (*n* = 60) at a 7:3 ratio using a complete randomization method.

### Data collection

General information of the children was recorded, including gestational age at birth (weeks), birth weight (g), gender (male/female), 5-min Apgar score, and maternal pregnancy complications; catheterization-related information, including age at catheterization (days), catheterization site (elbow/lower limb), catheter type (single-lumen/double-lumen), number of punctures, and qualification of the catheterization operator (primary/middle-senior); clinical information, including concurrent mechanical ventilation (yes/no), concurrent hypoproteinemia (yes/no), days of antibacterial drug use, and days of parenteral nutrition; laboratory indicators, including white blood cell count at catheterization ( × 10^9^/L), neutrophil percentage (%), C-reactive protein (mg/L), procalcitonin (ng/ml); catheter maintenance-related information, including catheter maintenance frequency (daily/non-daily).

### PICC catheterization and maintenance methods

Our hospital's NICU has 40 inpatient beds and employs 65 full-time neonatal nurses. The nurse-to-patient ratio is dynamically adjusted based on the clinical status of neonates: one nurse for every 1.5 stable neonates, and one nurse for every one critically ill neonate who requires continuous monitoring of vital signs (e.g., heart rate, respiration, blood pressure). All PICC catheterization operations were performed by certified neonatal nurses (with ≥3 years of NICU clinical experience and national PICC operation qualification certification) under ultrasound guidance (using a 7–12 MHz linear ultrasound probe) in strict accordance with the standard operating procedures for neonatal PICC insertion. The catheterization site was selected from peripheral veins with good visibility and patency, such as the elbow vein or the great saphenous vein of the lower limb. Catheter maintenance followed the unit's standardized protocol based on the 2009 Guidelines for Prevention of Catheter-Related Bloodstream Infections issued by the Infectious Diseases Society of America (IDSA) (9). Specific measures included: (1) Catheter flushing and sealing: 0.9% normal saline (1–2 ml per time) was used to flush the catheter before and after medication administration, and to seal the catheter after each use, to prevent blood clot formation and catheter blockage; (2) Dressing change: Sterile transparent polyurethane dressings were used, and changes were conducted every 72 h. If the dressing was soiled, wet, or loose, it was replaced immediately; (3) Skin disinfection: Before dressing change or catheter manipulation, 2% chlorhexidine gluconate was applied to the skin around the catheter insertion site (in a circular area with a diameter of ≥5 cm) in a spiral motion, and the skin was allowed to dry completely (for at least 30 s) before proceeding. All maintenance operations strictly adhered to the aseptic principle to reduce the risk of infection.

### Diagnostic criteria for CRBSI

The diagnostic criteria for CRBSI were those established by the IDSA: (1) The child showed infection manifestations such as fever (body temperature >38 °C) or hypothermia (body temperature < 36 °C), chills, and hypotension; (2) The same pathogen was isolated from both the peripheral blood culture and the catheter tip culture, and the colony count met certain criteria (colony count in peripheral blood culture ≥ 15 CFU, colony count in catheter tip culture ≥ 15 CFU); or although no pathogen was cultured, the child showed obvious infection manifestations, other infection foci were excluded, and the symptoms improved after catheter removal and antibacterial treatment. For culture-negative CRBSI cases, the diagnostic and classification process was standardized as follows, in line with the IDSA Guidelines: (1) Clinical symptom confirmation: The neonate exhibited specific infection signs, including persistent fever (>38 °C) or hypothermia (< 36 °C), unexplained hypotension (systolic blood pressure < 60 mmHg for term neonates, < 50 mmHg for preterm neonates), or lethargy (reduced spontaneous activity and poor response to tactile stimuli); (2) Microbiological testing: Peripheral blood samples were collected from two separate venipuncture sites for culture, and the catheter tip was cultured using the Maki roll technique (incubated at 37 °C for 48 h); all cultures showed no pathogenic growth; (3) Exclusion of alternative infections: Chest X-ray was performed to rule out pneumonia (no infiltrative shadows observed), urine culture (via sterile urine bag or suprapubic aspiration) to rule out urinary tract infection (no bacterial growth), and cerebrospinal fluid analysis (if neck stiffness or altered mental status was present) to rule out meningitis (normal white blood cell count and glucose level); (4) Therapeutic response evaluation: After catheter removal and administration of empirical broad-spectrum antimicrobials (e.g., ampicillin combined with cefotaxime), the neonate's infection symptoms completely resolved within 48–72 h, with no recurrence. In this study, 6 culture-negative CRBSI cases (three in the training set, three in the validation set) were classified using this process, accounting for 18.8% of all CRBSI cases.

### Development of the prediction model

In the training set, univariate analysis was first used to screen the possible influencing factors of CRBSI. Factors with *P* < 0.05 were included in the multi–factor Logistic regression analysis to screen out independent risk factors. The random forest algorithm was used to rank the importance of the screened influencing factors, and the variance inflation factor (VIF) was used for multicollinearity diagnosis to ensure that there was no severe multicollinearity among the variables. A nomogram model was constructed based on the screened independent risk factors. Each factor was scored, and the total score was calculated to predict the probability of CRBSI occurrence.

### Evaluation and validation of the prediction model

In the training set and the validation set, the receiver operating characteristic (ROC) curve and calibration curve were drawn to evaluate the predictive efficacy of the model, and the concordance index (C-index) was calculated. The Hosmer–Lemeshow test was used to evaluate the goodness-of-fit of the model. Decision curve analysis (DCA) was used to evaluate the clinical application value of the model to assist in clinical decision-making.

### Statistical analysis

The data were processed and analyzed using SPSS 26.0 statistical software and R 4.3.1 software. Count data were expressed as the number of cases and percentages, and the chi-square test or Fisher's exact probability method was used for comparison between groups. For measurement data conforming to the normal distribution, they were expressed as mean ± standard deviation (*x* ± s), and the *t*-test was used for comparison between groups. The random division of the sample into training and validation sets was implemented using the “sample” function in R language (version 4.3.1), following a complete randomization approach. The specific steps were: (1) Assign a unique numerical identifier (ranging from 1 to 200) to each of the 200 eligible neonates, corresponding to their medical record numbers; (2) Import the list of unique identifiers into R software; (3) Use the “sample” function with the parameter “size=140” and “replace=FALSE” to randomly select 140 identifiers without replacement, which were assigned to the training set; the remaining 60 identifiers were automatically included in the validation set. Univariate analysis and multivariate logistic regression analysis was used to screen independent risk factors, and a difference was considered statistically significant when *P* < 0.05. The “random Forest” package in R software was used for random forest analysis, the “rms” package was used to construct the nomogram model, the “p receiver operating characteristic” package was used to draw the ROC curve. The Bootstrap method was used for internal validation, the calibration curve was drawn, and the C-index was calculated. The “decision curve analysis. r” was used to perform decision curve analysis. For the training set (*n* = 140, 32 CRBSI cases), the events-per-variable (EPV) ratio was ~5.3 (32 events/6 predictors), and we used random forest variable screening and Bootstrap validation (1,000 resamples) to mitigate overfitting risk.

## Results

### Clinical characteristics between the training set and the validation set

A total of 215 neonates met the initial inclusion criteria, and 15 were excluded due to incomplete clinical data (*n* = 9) or transfer to other hospitals (*n* = 6), resulting in a final sample of 200. Among the 140 children in the training set, 32 cases (22.86%) had CRBSI; among the 60 children in the validation set, 14 cases (23.33%) had CRBSI. There was no statistically significant difference in the incidence of CRBSI between the two groups (*P* > 0.05). There were no statistically significant differences between the training set and the validation set in terms of all Clinical characteristics (all *P* > 0.05; Table 1).

**Table 1:** Comparison of clinical characteristics between the training set and the validation set.

**Indicators**	**Training set (*n* = 140)**	**Validation set (*n* = 60)**	***t*/χ^2^**	***P*-value**
Gestational age at birth (weeks)	32.56 ± 3.24	32.89 ± 3.56	0.641	0.522
Birth weight (g)	1,856.32 ± 456.78	1,889.56 ± 489.23	0.461	0.644
Gender (male/female)	78/62	33/27	0.009	0.925
5-min apgar score	8.56 ± 1.23	8.67 ± 1.15	0.591	0.555
Maternal pregnancy complications (yes/no)	35/105	17/43	0.242	0.062
Age at catheterization (days)	7.56 ± 3.21	7.89 ± 3.56	0.644	0.520
Catheterization site (elbow/lower limb)	97/43	42/18	0.010	0.919
Number of punctures	1.89 ± 0.76	1.95 ± 0.82	0.499	0.617
Combined with mechanical ventilation (yes/no)	56/84	22/38	0.196	0.657
Combined with hypoproteinemia (yes/no)	42/98	17/43	0.056	0.812
White blood cell count ( × 10^9^/L)	10.25 ± 3.56	10.56 ± 3.89	0.548	0.583
Neutrophil percentage (%)	63.25 ± 10.56	66.38 ± 11.23	1.884	0.061
C-reactive protein (mg/L)	8.56 ± 3.21	8.78 ± 3.56	0.429	0.667
Procalcitonin (ng/ml)	0.56 ± 0.23	0.58 ± 0.25	0.548	0.583
Qualification of catheterization operators	45/95	21/39	0.155	0.693
Catheter maintenance frequency (daily/non-daily)	60/80	25/35	0.024	0.875
Days of antimicrobial use	10.25 ± 4.56	10.56 ± 4.89	0.431	0.667
Days of parenteral nutrition	12.56 ± 5.21	12.89 ± 5.56	0.402	0.687

### Univariate analysis of risk factors for CRBSI in the training set

In the training set, the results of univariate analysis showed that there were statistically significant differences between the group with CRBSI and the group without CRBSI in terms of 5-min Apgar score, age at catheterization, catheterization site, number of punctures, combined mechanical ventilation, white blood cell count, C-reactive protein, qualification of the operator performing catheterization, days of antimicrobial drug use, and days of parenteral nutrition (all *P* < 0.05; Table 2).

**Table 2:** Univariate analysis of CRBSI in PICC-treated neonates in the NICU.

**Indicators**	**Occurrence of CRBSI group (*n* = 32)**	**No occurrence of CRBSI group (*n* = 108)**	***t*/χ^2^**	***P*-value**
Gestational age at birth (weeks)	32.07 ± 3.12	33.25 ± 3.05	1.912	0.057
Birth weight (g)	1,798.32 ± 356.78	1,956.56 ± 423.12	1.921	0.056
Gender (male/female)	18/14	60/48	0.005	0.944
5-min apgar score	7.87 ± 1.89	8.56 ± 1.05	2.663	0.008
Maternal pregnancy complications (yes/no)	12/20	23/85	3.456	0.063
Age at catheterization (days)	10.25 ± 3.56	8.56 ± 2.89	2.750	0.007
Catheterization site (elbow/lower limb)	13/19	84/24	16.011	0.001
Number of punctures	2.89 ± 0.96	1.56 ± 0.65	9.37	0.001
Combined with mechanical ventilation (yes/no)	18/14	38/70	4.564	0.032
Combined with hypoproteinemia (yes/no)	14/18	28/80	3.734	0.053
White blood cell count ( × 10^9^/L)	13.25 ± 4.56	10.94 ± 3.21	3.225	0.002
Neutrophil percentage (%)	75.25 ± 11.56	71.43 ± 10.23	0.800	0.740
C-reactive protein (mg/L)	15.25 ± 5.21	12.98 ± 3.56	2.826	0.005
Procalcitonin (ng/ml)	1.25 ± 0.56	1.14 ± 0.23	1.637	0.104
Qualification of catheterization operators	22/10	38/70	11.356	0.001
Catheter maintenance frequency (daily/non-daily)	14/18	46/62	0.013	0.907
Days of antimicrobial use	15.25 ± 5.56	12.56 ± 4.21	2.938	0.003
Days of parenteral nutrition	18.25 ± 6.21	14.73 ± 5.56	3.061	0.002

### Construction of the importance ranking of influencing factors for CRBSI complications in PICC children in the NICU based on the random forest algorithm

**Figure 1:**
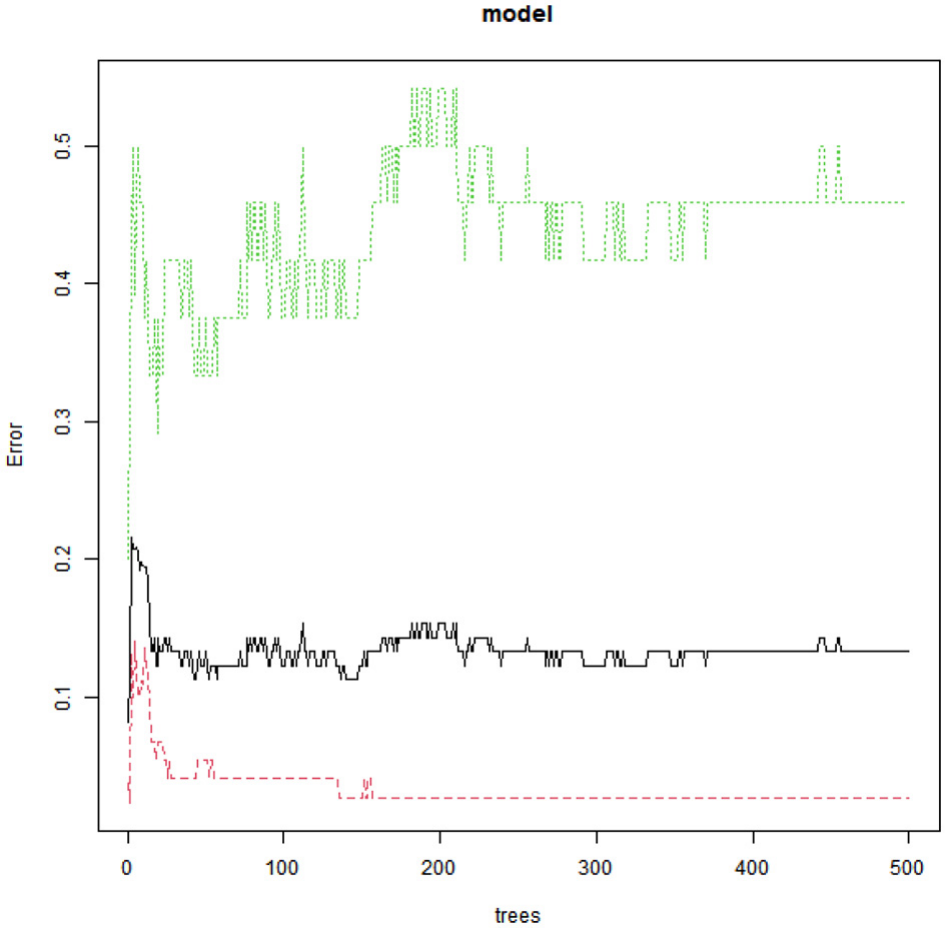
Relationship between model error rate and the number of decision trees.

**Figure 2:**
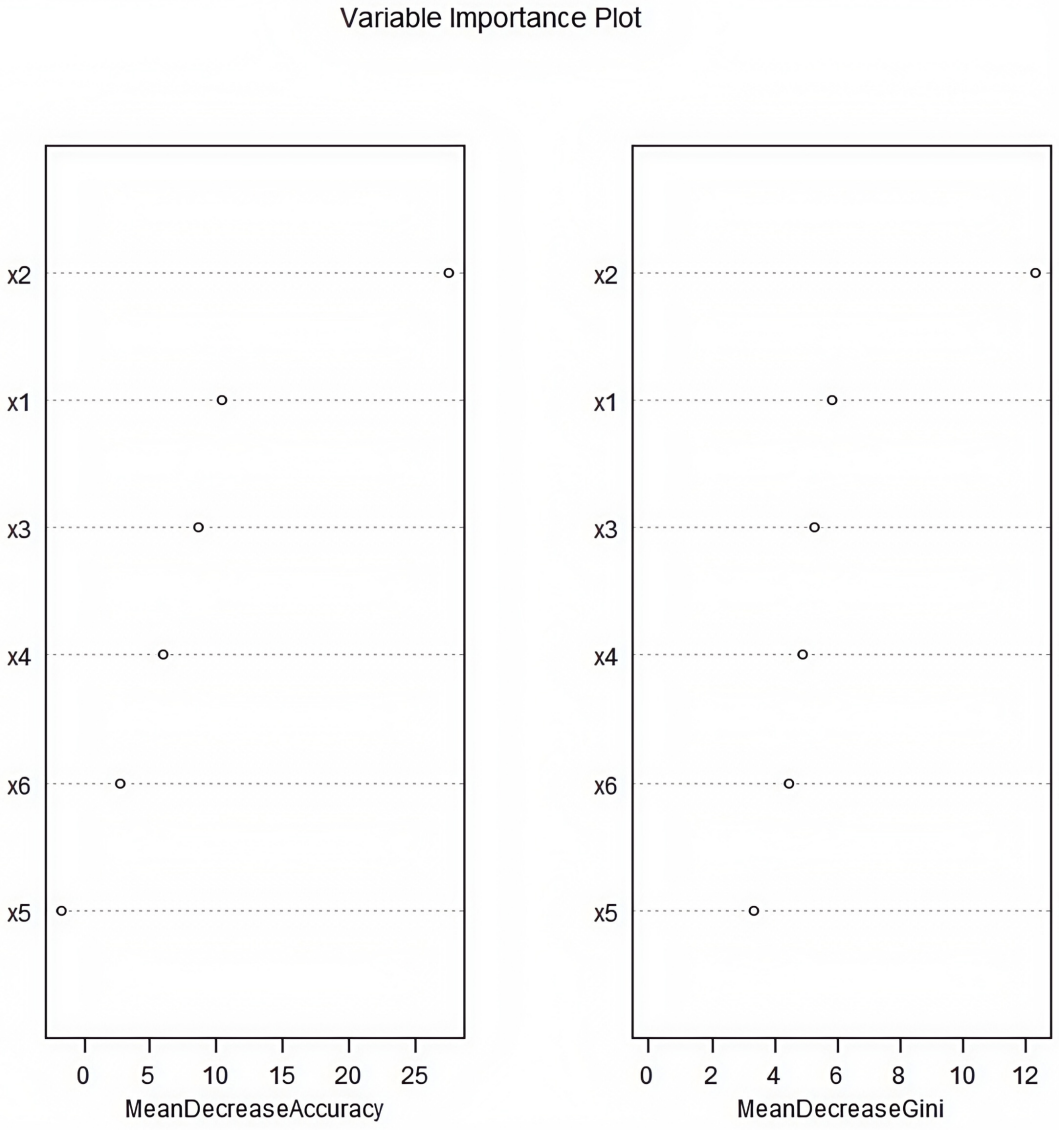
Importance ranking of influencing factors for catheter-related bloodstream infection in neonates with peripherally inserted central catheter patients (×1: 5-min Apgar score; ×2: Age of catheterization; ×3: Number of punctures; ×4: White blood cell count; ×5: Days of antimicrobial drug use; ×6: Days of parenteral nutrition).

A random forest model was constructed with the occurrence of CRBSI as the dependent variable (no occurrence = 0, occurrence = 1) and Apgar score, age at catheterization, number of punctures, white blood cell count, days of antimicrobial use, and days of parenteral nutrition as independent variables (Table 3). The obtained variable importance ranking was as follows: age at catheterization > 5-min Apgar score > number of punctures > white blood cell count > days of antimicrobial use > days of parenteral nutrition. Step-by-step random forest analysis was carried out according to the variable importance ranking. The results showed that the out-of-bag data error rate was the lowest when the number of variables was six. Therefore, these six variables were included in the random forest algorithm and multivariate Logistic regression to establish a prediction model. The average decrease in the variable Gini value was directly proportional to its importance in the model (Figures 1, 2).

**Table 3:** Variable assignment table.

**Variable**	**Meaning**	**Assignment**
X1	5-min apgar score	Continuous variable
X2	Age at catheterization	Continuous variable
X3	Number of punctures	Continuous variable
X4	White blood cell count	Continuous variable
X5	Days of antimicrobial use	Continuous variable
X6	Days of parenteral nutrition	Continuous variable
Y	Whether CRBSI occurred	No occurrence = 0, occurrence = 1

### Multivariate logistic regression analysis of risk factors for CRBSI in the training set

A multivariate Logistic regression analysis was conducted with the occurrence of CRBSI as the dependent variable (No occurrence = 0, Occurrence = 1) and the factors with *P* < 0.05 in the univariate analysis as covariates. The results showed that the age at catheterization, the number of punctures, the white blood cell count, the duration of antimicrobial use, and the duration of parenteral nutrition were independent risk factors for the occurrence of CRBSI (*P* < 0.05), while the 5-min Apgar score was an independent protective factor for the occurrence of CRBSI (*P* < 0.05). In the regression model, the tolerance of each variable was >0.1, the VIF was < 10, and the condition index was < 30. Moreover, there was no situation where the variance proportion of multiple covariates under the same eigenvalue was >50%, indicating that there was no collinearity among the covariates (Table 4).

**Table 4:** Multivariate logistic regression analysis of CRBSI in PICC-inserted neonates in the NICU.

**Indicators**	**β**	***SE***	***Wald***	***P*-value**	***OR***	**95% CI**
5-min apgar score	−0.553	0.211	6.868	0.009	0.575	0.381–0.870
Age in days at catheter placement	0.415	0.150	7.642	0.006	1.514	1.128–2.031
Number of punctures	1.329	0.426	9.727	0.002	3.779	1.639–8.713
White blood cell count	0.266	0.085	9.772	0.002	1.305	1.104–1.542
Days of antimicrobial use	0.158	0.074	4.555	0.033	1.172	1.013–1.255
Days of parenteral nutrition	0.142	0.051	7.714	0.005	1.152	1.043–1.274

### Development of a nomogram prediction model for CRBSI

**Figure 3:**
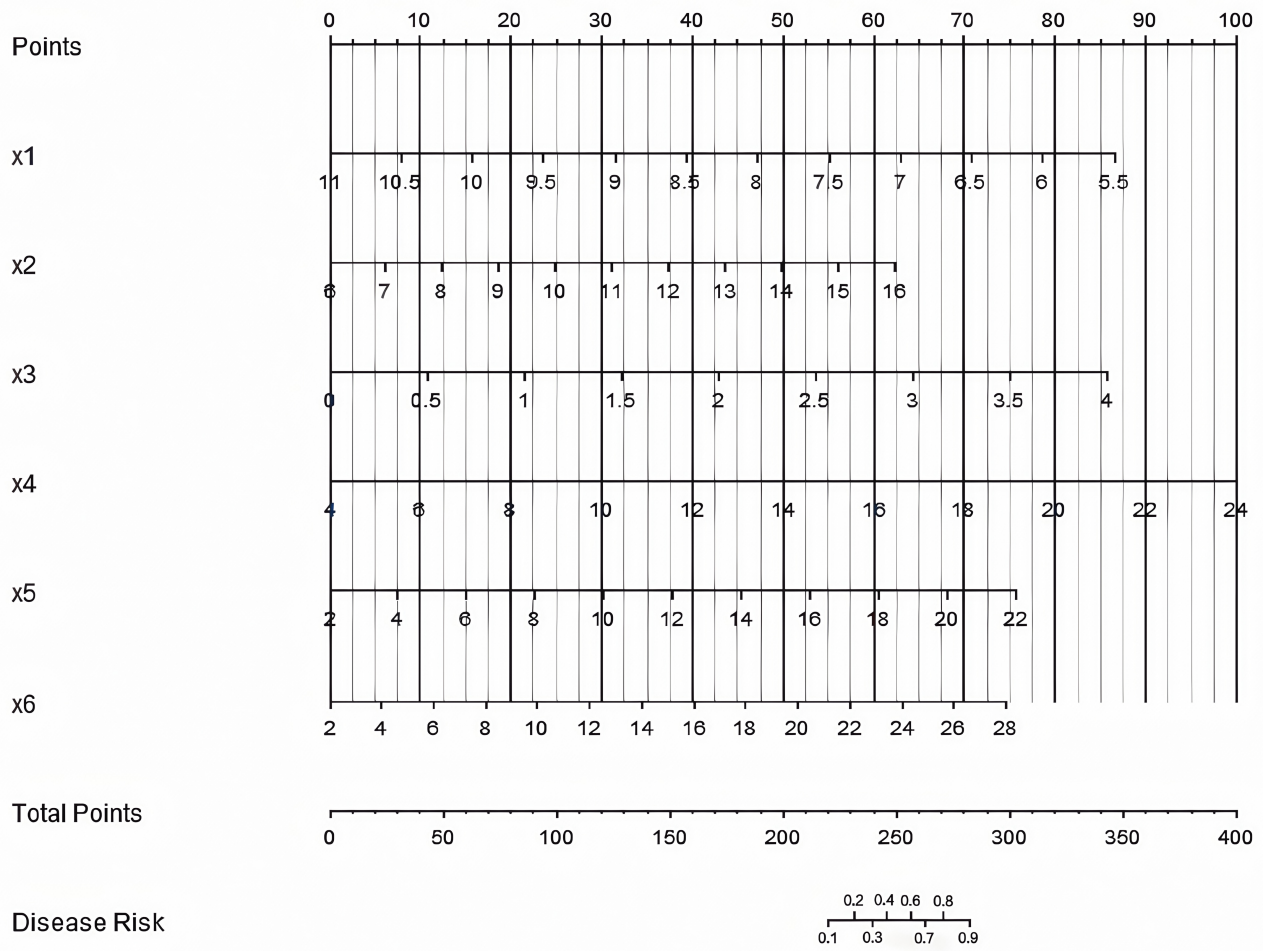
Nomogram prediction model for catheter-related bloodstream infection in neonates with peripherally inserted central catheter (×1: 5-min Apgar score, ×2 = Age of catheterization, ×3: Number of punctures, ×4: White blood cell count, ×5: Days of antimicrobial use, ×6: Days of parenteral nutrition. The “Points” axis (scale: 0–100 points) indicates the score assigned to each variable value; the “Total Points” axis (scale: 0–400 points) is the sum of scores from ×1 to ×6; the “Disease Risk” axis (scale: 0.0–1.0) represents the predicted probability of CRBSI based on the total score).

A nomogram prediction model was constructed based on the independent risk factors identified through multivariate Logistic regression analysis. Scores were assigned to each independent risk factor, and the total score for predicting the occurrence of CRBSI was calculated. To use the nomogram: (1) Locate the value of each variable on its corresponding axis; (2) Draw a vertical line upward to the “Points” axis to get the score for that variable; (3) Sum the scores of all variables to get the “Total Points”; (4) Draw a vertical line downward from “Total Points” to the “Disease Risk” axis to obtain the predicted CRBSI probability. The probability of CRBSI occurrence was reflected by this total score. The higher the total score, the higher the risk of CRBSI occurrence (Figure 3).

### Evaluation and validation of the CRBSI prediction model

**Figure 4:**
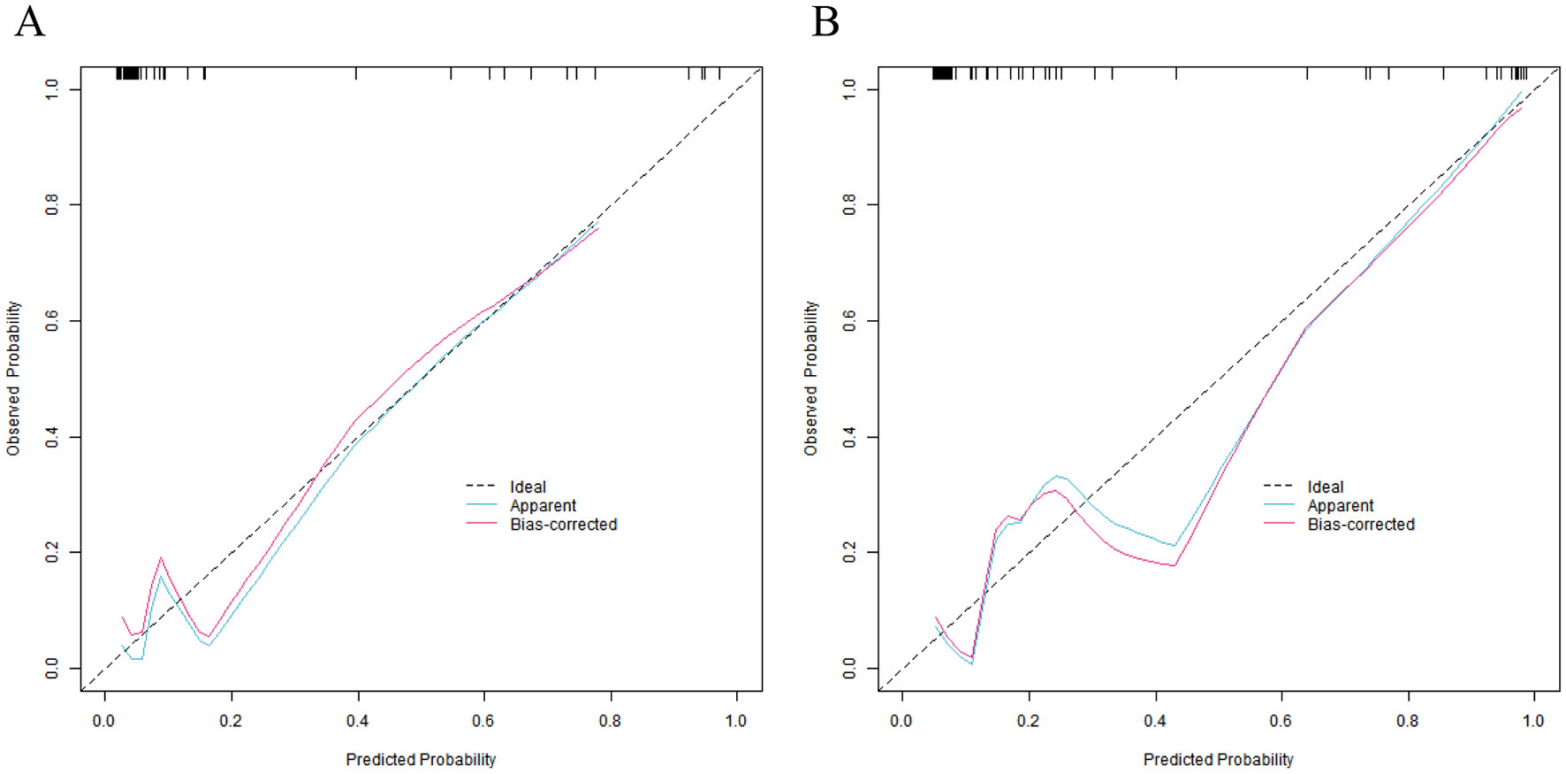
Calibration curves of nomogram prediction model for catheter-related bloodstream infection in neonates with peripherally inserted central catheter in the training set **(A)** and the validation set **(B)** (*X*-axis: Predicted CRBSI probability, *Y*-axis: Actual CRBSI probability).

**Figure 5:**
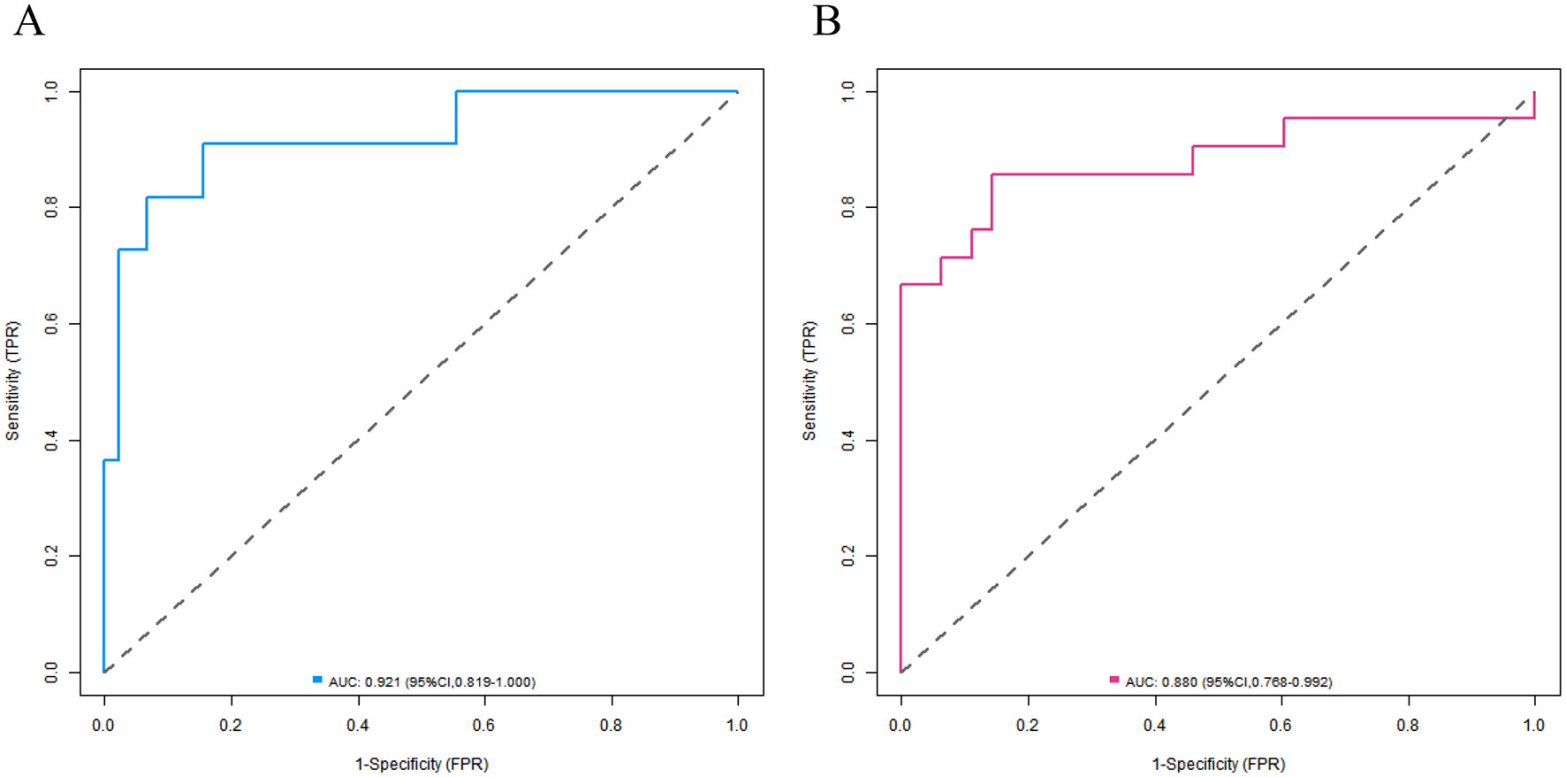
Receiver operating characteristic curves of nomogram prediction model for catheter-related bloodstream infection in neonates with peripherally inserted central catheter in the training set **(A)** and the validation set **(B)** (*X*-axis: 1—Specificity, *Y*-axis: Sensitivity).

In the training set and the validation set, the C-index values of the nomogram model were 0.923 and 0.881, respectively. The calibration curves showed a good agreement between the predicted values and the real values. The results of the Hosmer-Lemeshow test were χ^2^ = 6.696, *P* = 0.569 and χ^2^ = 4.590, *P* = 0.800, respectively. The ROC curves indicated that in the training set and the validation set, the area under the receiver operating characteristic curve (AUC) values of the nomogram model for predicting CRBSI were 0.921 (95% CI: 0.819–1.000) and 0.880 (95% CI: 0.768–0.992), respectively. The sensitivity and specificity were 0.909, 0.844 and 0.857, 0.857, respectively. The calibration curve is shown in Figure 4, and the ROC curve is shown in Figure 5.

### Decision curve analysis of the CRBSI prediction model

**Figure 6:**
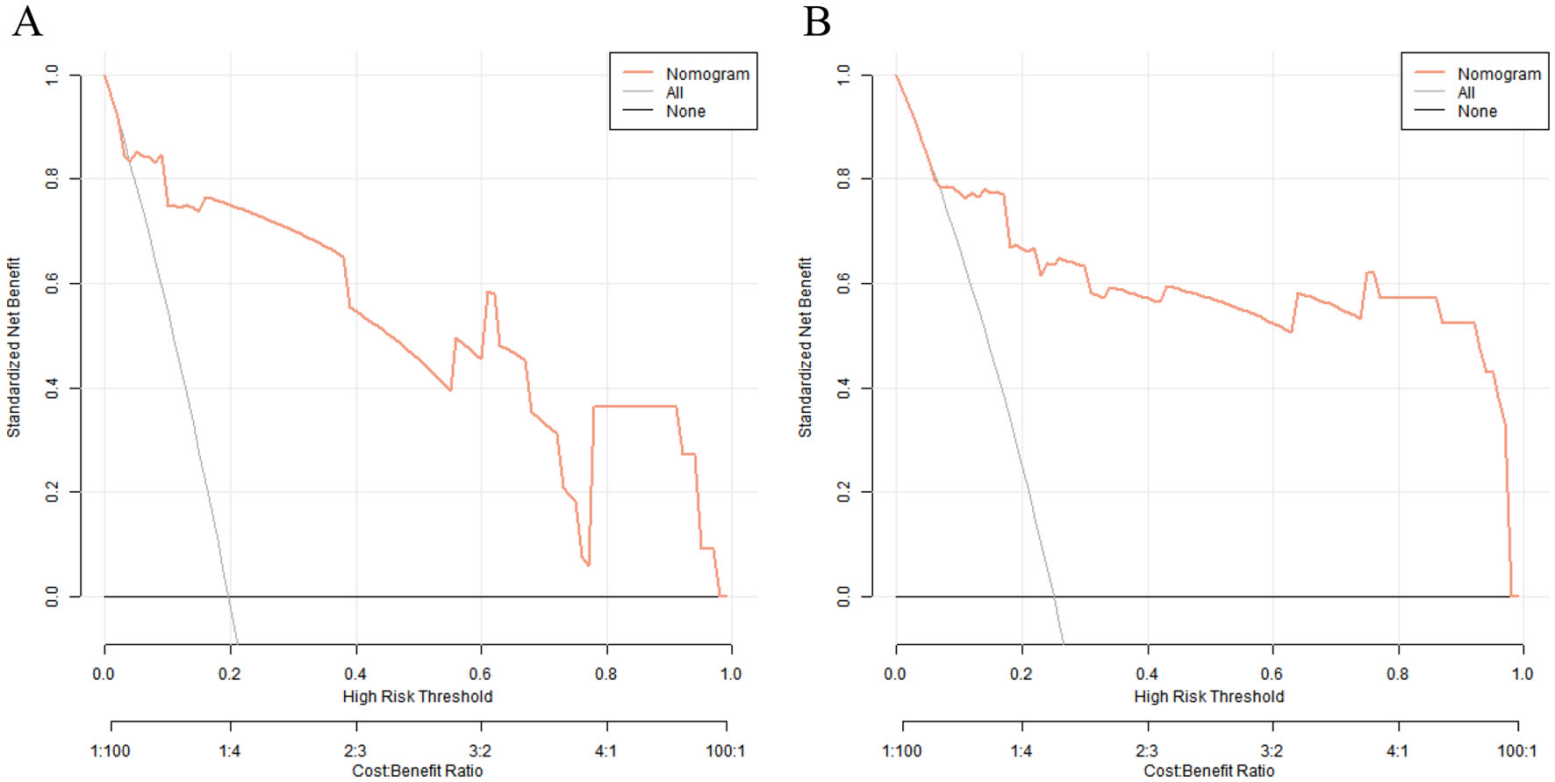
Decision curves of nomogram prediction model for catheter-related bloodstream infection in neonates with peripherally inserted central catheter in the training set **(A)** and the validation set **(B)** [*X*-axis: High Risk Threshold, *Y*-axis: Net Benefit, Nomogram: Net benefit of using the nomogram model to guide preventive measures, All: Net benefit of assuming all neonates develop CRBSI (initiating preventive measures for everyone), None: Net benefit of assuming no neonates develop CRBSI (no preventive measures)].

Decision curve analysis revealed that when the threshold probability ranged between 0.10 and 0.90, the decision to apply the nomogram model constructed in this study to predict the occurrence of CRBSI in PICC-treated infants in the NICU had more clinical benefits compared with the decisions of assuming that all infants would develop CRBSI or none of them would develop CRBSI before surgery (Figure 6).

## Discussion

In NICU, PICC has become an important route for nutritional support and treatment of critically ill newborns. However, CRBSI, as a severe complication of PICC, has always been a thorny problem in clinical practice (10). CRBSI not only prolongs the length of hospital stay and increases medical costs but also may lead to serious consequences such as sepsis and septic shock, and even endanger the life of newborns (11, 12). At present, the prevention and control of CRBSI in clinical practice mainly rely on standardized nursing measures, and there is a lack of precise risk assessment tools, making it difficult to achieve individualized prevention (13). In addition, the clinical manifestations of CRBSI in newborns are non-specific, and early identification is difficult. Therefore, it is urgent to establish a scientific prediction model to guide clinical intervention (14).

This study aimed to explore the influencing factors of CRBSI in newborns with PICC in the NICU and provide a basis for early clinical intervention by constructing a prediction model. The results showed that the incidence rates of CRBSI in the training set and the validation set were 22.86 and 23.33%, respectively, with no statistically significant difference between the two groups, indicating good homogeneity of the samples. Multivariate Logistic regression analysis screened out the age at catheterization, number of punctures, white blood cell count, days of antimicrobial use, and days of parenteral nutrition as independent risk factors, while the Apgar score was a protective factor. The variable importance ranking based on the random forest algorithm showed that the age at catheterization had the highest influence weight, followed by the Apgar score, number of punctures, etc. The further constructed nomogram model showed excellent predictive performance in the training set and the validation set, with C-indices of 0.923 and 0.881, respectively, and AUC of 0.921 and 0.880, respectively, suggesting that the model had high discrimination and accuracy. The following will conduct an in-depth analysis based on each key result and discuss the clinical value and limitations of the study.

The age at catheterization is the primary independent risk factor for the occurrence of CRBSI, and this finding is closely related to the physiological characteristics of newborns (15). Newborns with a younger age at catheterization, especially premature infants, have an immature immune system, low chemotactic and phagocytic functions of neutrophils, and insufficient complement system activity. Their ability to clear bacteria is significantly weaker than that of full-term infants (16). At the same time, the vascular conditions of low–age newborns are poor, which increases the difficulty of catheterization and may indirectly lead to an increased risk of catheter-related injury or contamination (17). In clinical practice, for extremely low-birth-weight infants or very premature infants, the necessity of catheterization should be fully evaluated before catheterization. Non-urgent catheterization should be postponed as much as possible, or a more minimally invasive catheterization method should be selected (18).

The number of punctures, as an independent risk factor, reflects the influence of catheterization operation techniques on the infection risk (19). Multiple punctures not only increase the probability of vascular endothelial injury and destroy the integrity of the vascular barrier but also may lead to repeated contact of the catheter surface with skin flora, increasing the chance of bacterial colonization. Studies have shown that when the number of punctures is ≥3, the risk of CRBSI can be increased by 2–3 times. This suggests that clinical practice needs to strengthen the skill training of catheterization operators and promote the ultrasound-guided catheterization technique to improve the success rate of single-puncture and reduce the infection risk (20). It is worth noting that the increase in the number of punctures may also be related to factors such as poor vascular conditions of newborns and insufficient experience of operators. Therefore, comprehensive consideration should be given in combination with the individual situation of the newborn and the operation level.

An elevated white blood cell count is an important early warning indicator of CRBSI. The Odds Ratio (OR) indicates that for every 1 × 10^9^/L increase in the peripheral white blood cell level, the infection risk increases significantly. This is directly related to white blood cells being the core component of the body's immune response: the systemic inflammatory response caused by bacterial invasion can lead to an increase in the white blood cell count, and the formation of biofilm on the surface of the PICC catheter and bacterial entry into the blood further exacerbate this process (21). It is worth noting that newborns, especially premature infants, may have a decreased rather than increased white blood cell count in case of severe infection. Therefore, clinical practice needs to comprehensively judge in combination with inflammatory indicators such as C-reactive protein and procalcitonin.

The days of antimicrobial use and the days of parenteral nutrition, as independent risk factors, reveal the influence of iatrogenic factors on CRBSI. Long-term use of antimicrobial agents can destroy the normal intestinal flora, lead to excessive proliferation of drug-resistant bacteria, and increase the risk of catheter colonization. When providing parenteral nutrition support, hypertonic nutrient solutions provide a good culture medium for bacterial growth, and long-term intravenous nutrition may lead to weakening of the intestinal mucosal barrier function and increase the risk of endotoxin translocation (22). In clinical practice, the principle of “precise medication” should be followed to avoid extending the course of antimicrobial agents without indications, and enteral nutrition should be initiated as early as possible to reduce the infection risk.

The Apgar score, as a protective factor, shows that the higher the score, the lower the risk of CRBSI according to its OR value. This result conforms to clinical logic: the Apgar score reflects the degree of asphyxia at birth. Newborns with a low score often have multi-organ functional damage, especially suppression of the immune system, while newborns with a normal score have stronger compensatory ability and a more perfect defense mechanism against infection (23). Therefore, for high-risk newborns with a low Apgar score, infection monitoring should be strengthened after catheterization, and more strict preventive measures should be taken.

This study used the random forest algorithm to rank the importance of risk factors. The results showed that age at catheterization > Apgar score > number of punctures > white blood cell count > days of antimicrobial use > days of parenteral nutrition. This ranking complements the results of Logistic regression, highlighting the unique value of different analysis methods in variable screening. As a non-parametric machine learning method, the random forest can effectively handle the complex interaction between variables. Its importance assessment based on the reduction of Gini impurity provides a more accurate quantitative basis for clinical identification of key risk factors.

The age at catheterization ranked first in both analysis methods, further confirming the strong correlation between the age of newborns and the risk of CRBSI. Clinically, a “stratified management by age” strategy can be established based on this: for newborns with an age at catheterization < 72 h, the highest-level infection prevention and control measures should be implemented, such as daily assessment of the necessity of the catheter and increasing the frequency of dressing changes. The high weight of the Apgar score suggests that the degree of asphyxia at birth is an important baseline indicator for predicting later-stage CRBSI, which provides a new basis for risk stratification at the time of admission to the NICU. The high ranking of the number of punctures emphasizes the urgency of standardizing operation techniques. The hospital infection management department can include the success rate of single-puncture in the assessment indicators of catheterization operators to promote operation standardization.

The nomogram model constructed in this study showed excellent predictive performance in the training set and the validation set, with C-indices of 0.923 and 0.881, respectively, far exceeding the clinically acceptable threshold of 0.7, indicating that the model has a strong ability to distinguish between cases with and without CRBSI. To address potential overfitting, we calculated the EPV ratio (≈5.3) for the training set and implemented two risk-mitigation measures: (1) Random forest algorithm-based variable screening to exclude weakly associated predictors, retaining only six core variables; (2) Bootstrap internal validation (1,000 resamples), which adjusted the training set C-index to 0.912 (from 0.923) while preserving the validation set C-index at 0.881. This confirms the model's stability despite the modest sample size. The AUC of the ROC curve was 0.921 in the training set and 0.880 in the validation set, and both the sensitivity and specificity exceeded 0.85, suggesting that the model can effectively identify high-risk newborns and reduce the misdiagnosis rate in clinical application. The calibration curve showed a high degree of agreement between the predicted probability and the actual probability, and the *P* value of the Hosmer–Lemeshow test was > 0.05, confirming that the model had a good goodness of fit and the prediction results were reliable.

DCA showed that when the threshold probability was in the range of 0.1–0.9, the clinical net benefit of using the nomogram model was significantly higher than the decision-making strategies of “CRBSI occurs in all the population” or “CRBSI does not occur in all the population.” This means that clinicians can accurately formulate prevention and control measures based on the individual risk probability calculated by the model: for newborns with a risk probability >0.7, shortening the catheter indwelling time and increasing the frequency of blood culture monitoring can be considered; for newborns with a risk probability < 0.3, the monitoring intensity can be appropriately reduced to optimize the allocation of medical resources.

The visual design of the nomogram further improves the clinical practicability of the model. By assigning corresponding scores to each risk factor, doctors can quickly calculate the total score at the bedside and convert it into the probability of CRBSI occurrence. For example, for a newborn with an age at catheterization < 3 days, number of punctures ≥3, and white blood cell count >15 × 10^9^/L, the total score may exceed the critical value, indicating that an enhanced prevention plan should be initiated. This “quantitative decision-making” model helps to reduce the bias caused by subjective experience and promotes the prevention and control of CRBSI toward precision medicine. To translate the model's statistical performance into meaningful clinical benefit, we have developed a streamlined clinical application protocol: the model should be applied at two key stages of neonatal care—within 24 h of PICC insertion (to enable early identification of high-risk neonates and timely initiation of preventive measures before potential infection) and weekly during PICC indwelling (to reassess risk as clinical status changes, such as prolonged antimicrobial use, increased white blood cell count, or deteriorated nutritional status, ensuring interventions remain up-to-date); based on the model's predicted CRBSI probability, three risk tiers with matching preventive measures are proposed: high risk (predicted probability >70%) involves daily catheter necessity assessment (prompt removal of unnecessary catheters), daily peripheral blood culture monitoring (early infection detection), 48-h dressing change intervals (reduced bacterial colonization), and infectious disease specialist consultation (individualized antimicrobial regimens based on local resistance patterns); moderate risk (30%−70%) includes twice-weekly catheter necessity assessment, 72-h standard dressing changes (with immediate replacement if soiled/wet/loose), and daily vital sign monitoring (focus on temperature fluctuations >38 or < 36 °C, heart rate, and respiration to detect early infection); low risk (< 30%) requires only weekly catheter necessity assessment and basic maintenance (72-h dressing changes, routine flushing) to avoid over-medicalization; additionally, to improve bedside usability, we plan to collaborate with our hospital's information technology department to integrate the nomogram into the electronic medical record (EMR) system, where the EMR will automatically extract the six predictors (5-min Apgar score, age at catheterization, number of punctures, white blood cell count, days of antimicrobial use, days of parenteral nutrition) from the patient's record, calculate real-time total risk scores and CRBSI probabilities, and display corresponding preventive measures, eliminating manual calculation errors and promoting standardized model application across the NICU.

The core advantage of this study lies in the integration of traditional statistical methods and machine learning algorithms, forming a complete research chain of “multi-dimensional analysis-precise modeling-clinical verification.” Compared with studies using only Logistic regression, this study ranks the importance of risk factors through the random forest algorithm, which can more comprehensively capture the non-linear relationships and interactions between variables and avoid the bias that may be caused by a single method. For example, the interaction between the Apgar score and the age at catheterization may affect the susceptibility of newborns to infection, and this complex relationship is more fully reflected in the random forest analysis (24).

In addition, the indicators included in the study are both clinically practical and accessible. For example, the age at catheterization, number of punctures, and Apgar score are all routinely recorded data in the NICU, without additional detection costs, which lays a foundation for the popularization and application of the model in primary hospitals. Compared with previous studies, this study for the first time included the Apgar score as a protective factor in the prediction model, enriching the dimensions of CRBSI risk assessment and providing new evidence for the association between the birth status and the later-stage infection risk.

Although this study has achieved valuable results, there are still several limitations, and their potential impacts on the study results and model clinical applicability are discussed as follows: Firstly, the study was a single-center retrospective design, and the samples are from the NICU of the same hospital, which may have selection bias and limit the extrapolation of the study results. To address the issue of external generalizability, our team has developed plans for a multicenter prospective validation study. We intend to collaborate with three tertiary maternal and child health hospitals in Jiangxi, Guangdong, and Hunan provinces, and plan to enroll neonates with PICC admitted between January 2025 and December 2026. This upcoming study will further verify the model's predictive efficacy across different clinical practice protocols and patient populations, aiming to enhance its applicability in diverse NICU settings. Secondly, the study did not include variables such as neonatal illness severity scores (Neonatal Critical Illness Score, NCIS) and detailed operator experience (years of PICC operation). The omission of NCIS may reduce the model's accuracy in predicting CRBSI for critically ill neonates (who have more severe immune dysfunction), while the lack of operator experience years may lead to underestimation of risk for neonates treated by less experienced operators (with higher puncture-related vascular injury rates). These variables will be included in future studies to optimize the model. Thirdly, the sample size was relatively small (*n* = 200), with an EPV ratio of ~5.3 in the training set—lower than the ideal EPV ≥10. This may result in slight overestimation of the model's discriminative ability (the training set AUC of 0.921 might be marginally higher than the true value). However, we mitigated this impact through Bootstrap validation (1,000 resamples), which adjusted the training set C-index to 0.912 (from 0.921), and the validation set C-index (0.880) remained stable, confirming the model's core predictive reliability. A larger multicenter sample (*n* > 500) will be used in future studies to further minimize overfitting. Fourthly, variables related to catheter care compliance (e.g., hand hygiene adherence, dressing change timeliness) were not recorded in medical records. Poor hand hygiene is a known CRBSI risk factor, but our unit's monthly quality monitoring (via direct observation and hand sanitizer consumption data) shows a hand hygiene adherence rate of >95%, and a dressing change timeliness rate of >98%. Thus, the impact of unrecorded compliance on CRBSI incidence and model performance is considered minimal. Fifthly, the model does not include catheter care protocol variables such as antiseptic type and dressing material. Our center uses 2% chlorhexidine gluconate and transparent polyurethane dressings, but units using other antiseptics (e.g., povidone-iodine) or dressings (e.g., gauze) may have different CRBSI risks. This limits the model's applicability in such settings, and future multicenter studies will incorporate these variables to enhance generalizability. Additionally, the study did not include microscopic indicators such as catheter tip culture results and biofilm detection, making it difficult to reveal the occurrence mechanism of CRBSI at the molecular level. In future multicenter studies, we will also supplement key variables including the NCIS, operators' years of PICC insertion experience, and pathogen types from catheter tip cultures to further improve the model's predictive precision.

Future studies can be expanded in the following directions: First, explore the association between gene polymorphisms and the susceptibility to CRBSI. For example, Toll-like receptor 4 (TLR4) gene polymorphisms may affect the immune response of newborns to bacterial infections. Incorporating molecular biology indicators into the prediction model can improve the prediction accuracy. Second, develop a dynamic prediction model. Combine real-time monitored inflammatory indicators such as the dynamic change of PCT to update the risk probability and achieve real-time early warning of the infection risk. Third, conduct intervention studies based on the prediction model to verify whether risk-stratified management can truly reduce the incidence of CRBSI and provide evidence-based support for the clinical translation of the model.

This study constructed an efficient CRBSI prediction model through multivariate analysis and machine learning methods, confirming that indicators such as the age at catheterization and the number of punctures is key factors affecting neonatal PICC-related CRBSI. The high predictive performance of the nomogram model provides a practical risk assessment tool for clinical practice, which helps to achieve precise prevention and control of CRBSI. In clinical practice, stratified intervention strategies can be formulated based on the model results. Enhanced prevention measures should be implemented for high-risk newborns, while over-medicalization of low-risk newborns should be avoided. In the future, multi-center studies and molecular biology exploration are needed to further improve the model and promote the improvement of the prevention and control level of neonatal CRBSI.

## Data Availability

The original contributions presented in the study are included in the article/Supplementary material, further inquiries can be directed to the corresponding author.
